# Breeding of Penta Null Soybean [*Glycine max* (L.) Merr.] for Five Antinutritional and Allergenic Components of Lipoxygenase, KTI, Lectin, 7S α′ Subunit, and Stachyose

**DOI:** 10.3389/fpls.2022.910249

**Published:** 2022-06-07

**Authors:** Sang Woo Choi, Sarath Ly, Jeong Hwan Lee, Hyeon Su Oh, Se Yeong Kim, Na Hyeon Kim, Jong II Chung

**Affiliations:** Department of Agronomy, Gyeongsang National University, Jinju, South Korea

**Keywords:** penta null, lipoxygenase, KTI, lectin, 7S α′ subunit, stachyose

## Abstract

Soybean [*Glycine max* (L.) Merr.] is an excellent source of protein, oil, carbohydrates and many other bioactive ingredients for humans. However, several antinutritional and allergenic components such as lipoxygenase, KTI, lectin, 7S α′ subunit, and stachyose exist in the raw mature seed. Genetic removal of these components would be the best method to improve soybean food quality. The objectives of this research were to breed a new soybean line with penta null recessive alleles (*lox1*/*lox1/lox2*/*lox2/lox3*/*lox3*-*ti*/*ti*-*le*/*le*-*cgy*1/*cgy1*-*rs2*/*rs2*) for these five components and to evaluate agronomic traits for a breeding line with penta null alleles. Seven germplasms were used to breed the penta null strain. Analysis of lipoxygenase, KTI, lectin, 7S α′ subunit, and stachyose components in mature seeds was conducted by SDS-PAGE, western blot, and HPLC. One breeding line with penta null recessive alleles was developed. The breeding line has purple flowers, tawny pubescence, a determinate growth habit, and light yellow pods at maturity. The seed of the breeding line has a yellow hilum and yellow seed coat color. The stem height of the breeding line was 53.0 cm. The stachyose content of the breeding line was 2.9 g/kg. The 100-seed weight of the breeding line was 31.1 g and yield (t/ha) was 2.80. This is the first soybean strain with the penta null (*lox1lox2lox3*/*lox1lox2lox3*-*ti*/*ti*-*le*/*le*-*cgy1*/*cgy1*-*rs2/rs2*) genotype (free of lipoxygenase, KTI, lectin, and 7S α′ subunit proteins, and with low stachyose content).

## Introduction

Soybean [*Glycine max* (L.) Merr.] is a major leguminous crop that has been cultivated for thousands of years. Soybean seeds are an important source of protein, oil, carbohydrates, isoflavones, and many other nutrients for human food and animal feed. Generally, soybean seeds contain about 40% protein, 20% oil, 30% carbohydrates, and various health functional ingredients. However, there are also antinutritional and harmful immunological components such as lipoxygenase protein, Kunitz trypsin inhibitor (KTI) protein, 7S α′ subunit protein, and stachyose in mature soybean seeds.

Lipoxygenase protein causes undesirable grassy and beany flavors in foods containing soybean due to the oxidation of polyunsaturated fatty acids. There are three lipoxygenases (*Lox1*, *Lox2,* and *Lox3*) in mature seeds. Previous studies demonstrated that the absence of each enzyme is under the control of three null alleles, *lox1*, *lox2*, and *lox3*, which are inherited as simple recessive alleles (Hildebrand and Hymowitz, 1982; Kitamura et al., 1983; Davies and Nielsen, 1986).

Lipoxygenase-free genotypes are better accepted due to the production of very low levels of hexanal compounds (Kobayashi et al., 1995). The development of lipoxygenase-free genotypes through genetic elimination is the key to removing the beany flavor. Several lipoxygenase protein-free cultivars have been improved (Kim et al., 1997, 2015; Chung, 2014). Recently, lipoxygenase-free mutants were obtained using a pooled CRISPR-Cas9 system (Wang et al., 2020). Soybean Kunitz trypsin inhibitor (KTI) protein, which was isolated and crystallized by Kunitz (1945), is a small and non-glycosylated protein possessing 181 amino acid residues with 21.5 kDa. KTI protein severely inhibits trypsin, thus reducing food intake by diminishing digestion and absorption. Orf and Hymowitz (1979) have identified two soybean accessions (PI157440 and PI196168) lacking the KTI protein in the USDA Soybean Germplasm Collection. The presence of KTI protein was controlled at a single locus with a codominant multiple allelic series that identified a recessive allele designated *ti* that lacks the KTI protein (Orf and Hymowitz, 1979). Crude protein from the *titi* genotype showed a 30%–50% reduction in trypsin inhibitor activity compared with the *TiTi* genotype. The *Ti* gene was found to be on chromosome 8. Soybean with normal protein content was developed through a marker-assisted survey of 180 recombinant inbred lines (RILs) developed from the cross KTI + parent and KTI-free parent KTI-free (Rani et al., 2020). A breeder-friendly Kompetitive Allele-Specific PCR (KASP) marker linked to low Kunitz trypsin inhibitor (KTI) in soybean seeds has been developed (Rosso et al., 2021). A KTI-free vegetable soybean genotype was developed using *Ti-*linked simple sequence repeat (SSR) markers (Kumar et al., 2021). Recently, Lox-2 and KTI-free soybean lines were developed by introgressing null alleles of Lox-2 and KTI genes in the variety “JS97-52” through marker-assisted backcross breeding (Kumar et al., 2022). Soybean lectin protein with 120 kDa molecular weight is a major antinutritional element and can strongly inhibit degradation by proteases under *in vitro* and *in vivo* conditions (Pull et al., 1978). Consuming food including lectin protein can cause symptoms such as nausea, vomiting, and diarrhea (Katsuya et al., 2007). Soybean seed lectin is controlled by a single gene designated *Le* (*le*) and the *lele* genotype results in a lack of lectin in mature seed (Orf et al., 1978). The *Le* gene was found to be on chromosome 2. The two main seed storage proteins are glycinin (11S) and β-conglycinin (7S), which constitute about 70% of storage protein (Krishnan et al., 2009). Almost 90% of 7S protein is accumulated by β-conglycinin, which has three subunits (α, α′, β), and these three subunits are the major allergens of soybean protein (Medic et al., 2014). The three subunits of β-conglycinin (7S), α, α′, and β are dominated by genes *Cgy1*, *Cgy2*, and *Cgy3*, respectively (Davies et al., 1985). Among these three genes, a line with the homozygous recessive *Cgy1* gene cannot produce the 7S α′ subunit protein in mature seed (Kitamura et al., 1984). *Cgy1* gene was found to be on chromosome 10. A soybean experimental line (BSH-3) that is 7S α′ subunit protein free was developed by crossing the mutant donor line “HS99B” with the Chinese cultivar “Dongnong47” (Song et al., 2018).

Raffinose and stachyose are considered antinutritional factors because humans cannot digest them after absorption (Hata et al., 1991). Stachyose is the primary carbohydrate in soybean seed. Stachyose content ranges from 14 to 41 g/kg on a dry weight basis and is environmentally stable but genotypically dependent (Hymowitz et al., 1972). The amount of stachyose was controlled by a single gene or by a major quantitative trait locus (QTL; Skoneczka et al., 2009). The raffinose synthase 2 gene is a pathway for raffinose and stachyose biosynthesis. Soybean line PI200508 with a homozygous recessive genotype (*rs2rs2*) showed low raffinose and stachyose content (Dierking and Bilyeu, 2008). *RS2* locus was found to be located on chromosome 6. Two SSR markers, Sat_293 (LG-K/*chr*9) and Satt281 (LG-C2/*chr*6), were identified for stachyose in two F_2_ populations (Jha et al., 2022).

Because of these antinutritional factors and allergens that exist in raw soybean seeds, heat treatment or other methods are needed to eliminate or reduce these components and secure the efficiency of nutrient absorption and food safety. But these treatments cause some changes and reduce soybean quality (Chen et al., 2019). Also, heat inactivation of the lipoxygenase at an industrial level not only incurs extra cost but also affects the solubility and functionality of proteins (MaCleod and Ames, 1988). Antigenic proteins remain in soybean food even after heat treatment and fermentation (Wilson et al., 2008). Genetic removal of lipoxygenase, KTI, lectin, 7S α′ subunit, and stachyose components that exist in mature soybean seed would be the best method for the soybean food industry. Only a few papers on soybean free of these antinutritional and allergenic components have been published. A soybean line with a triple null recessive genotype (*ti*/*ti*-*le*/*le*-*p34*/*p34*) for KTI, lectin, and P34 proteins was developed (Schmidt et al., 2015). A soybean line with a tetra null recessive genotype (*lox1lox2lox3*/*lox1lox2lox3*-*ti*/*ti*-*le*/*le*-*cgy*1/*cgy1*) for lipoxygenase, KTI, lectin, and 7S α′ subunit proteins was also developed (Choi et al., 2021). So far, a soybean line with a penta null recessive genotype for antinutritional and allergenic factors has not been developed. Therefore, the objectives of this research were to breed a new soybean line with a yellow seed coat color and penta null recessive alleles (*lox1lox2lox3*/*lox1lox2lox3*-*ti*/*ti*-*le*/*le*-*cgy*1/*cgy1*-*rs2*/*rs2*) for lipoxygenase, KTI, lectin, 7S α′ subunit, and stachyose components and to evaluate agronomic traits for a breeding line with penta null alleles.

## Materials and Methods

### Breeding Materials

Seven germplasms were used to improve the new soybean strain with the penta null genotype for five components. The presence and absence of four proteins, stachyose content, seed coat, 100-seed weight (g), and origin of the seven germplasms used in this study are presented in Table 1. Three breeding lines (*lox1lox1*/*lox2lox2*/*lox3lox3* genotype—lipoxygenase protein free; *lox1lox1*/ *lox2lox2*/*lox3lox3-cgy1* genotype—lipoxygenase and 7S α′ subunit proteins free; *lox1lox1*/*lox2lox2*/*lox3lox3*-*ti*-*le*-*cgy1* genotype—lipoxygenase, KTI, lectin and 7S α′ subunit proteins free, Choi et al., 2021) and one germplasm (PI200508) were used to create a genetic population. The PI200508 parent has an *rs2rs2* genotype with low stachyose content (Dierking and Bilyeu, 2008). Three breeding lines and one germplasm have a yellow seed coat color in mature seeds.

**Table 1:** Seed coat, 100-seed weight, stachyose content, origin, presence or absence of lipoxygenase, Kunitz trypsin inhibitor (KTI), lectin and 7S α′ subunit for seven germplasms.

Germplasm name	Lipoxygenase			KTI	Lectin	7S α′ subunit	Stachyose	Seed coat	100-seed weight (g)	Origin
	1	2	3							
PI408251	Absent	Present	Present	Present	Present	Present	Normal	Black	6.1	Korea
PI86023	Present	Absent	Present	Present	Present	Present	Normal	Green	16.8	Japan
PI417458	Present	Present	Absent	Present	Present	Present	Normal	Yellow	11.8	Japan
PI200508	Present	Present	Present	Present	Present	Present	Low	Yellow	13.1	Japan
PI506876	Present	Present	Present	Present	Present	Absent	Normal	Yellow	15.1	Japan
PI157440	Present	Present	Present	Absent	Present	Present	Normal	Yellow	14.5	Korea
T102	Present	Present	Present	Present	Absent	Present	Normal	Black	6.7	United States

### Breeding Scheme

**Figure 1:**
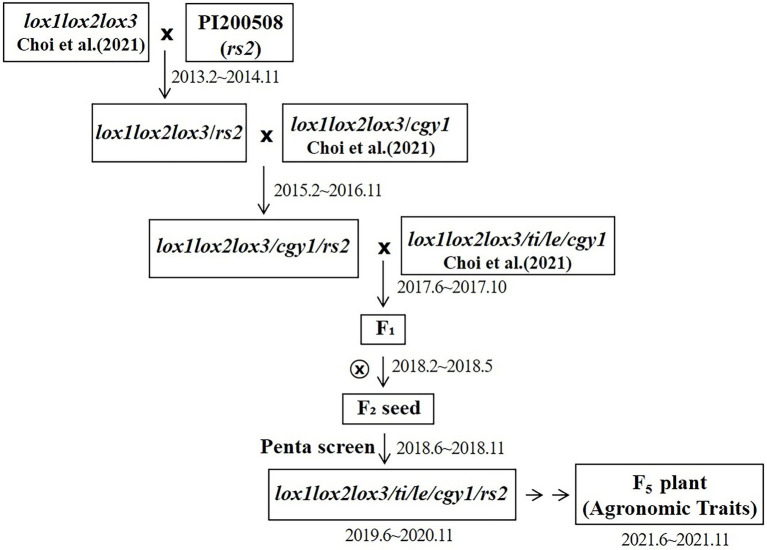
Scheme for the development of penta null alleles (*lox1lox2lox3*/*lox1lox2lox3*-*ti*/*ti*-*le*/*le*-*cgy1*/*cgy1-rs2rs2*) for lipoxygenase, KTI, lectin, 7S α′ subunit and stachyose components.

The parent with the *lox1lox2lox3/lox1lox2lox3* genotype was crossed with the PI200508 parent with the *rs2rs2* genotype to select a seed with a homozygous *lox1lox2lox3*/*lox1lox2lox3*-*rs2rs2* genotype. *lox1lox2lox3* alleles were determined by identifying the absence of lipoxygenase protein using SDS electrophoresis. The *rs2rs2* genotype, which determines the low stachyose content, was identified by DNA marker. A plant with a *lox1lox2lox3/lox1lox2lox3*-*rs2/rs2* genotype (lipoxygenase protein free and with low stachyose content) was developed. The plant was then crossed with the plant with the *lox1lox2lox3/lox1lox2lox3*-*cgy1/cgy1* genotype (lipoxygenase and 7S α′ subunit proteins free). The *cgy1cgy1* genotype was determined by identifying the absence of 7S α′ subunit protein using SDS electrophoresis. From this cross, a triple null genotype (*lox1lox2lox3*/*lox1lox2lox3*-*rs2*/*rs2*-*cgy1*/*cgy1*, free of lipoxygenase and 7S α′ subunit proteins, and with low stachyose content) was developed. During the summer of 2017, F_1_ pollinations between *lox1lox2lox3*/*lox1lox2lox3*-*rs2*/*rs2*-*cgy1*/*cgy1* parent and *lox1lox2lox3* /*lox1lox2lox3*-*ti*/*ti*-*le*/*le*-*cgy1/cgy1* parent were made in a greenhouse to produce seeds possessing penta null alleles (*lox1lox2lox3*/ *lox1lox2lox3*-*ti*/*ti*-*le*/*le*-*cgy1*/*cgy1*-*rs2*/*rs2*). When mature, the hybrid seed borne on a female parent was collected and hand threshed. The resultant F_1_ seeds were planted in the greenhouse on 25 February 2018. F_1_ hybridity was confirmed based on pod color (i.e., tan female mated to brown male; the female self is tan, the F_1_ hybrid is brown). All F_1_ plants were individually harvested and were bulked after the confirmation of hybridity. A total of 172 F_2_ seeds were obtained. *Titi* and *lele* genotypes were determined by identifying the absence of KTI and lectin proteins using the western blot technique. Each seed was analyzed to screen for genotypes with tetra recessive alleles [*lox1lox2lox3*/*lox1lox2lox3*-*ti*/*ti*-*le*/*le*-*cgy1*/*cgy1* (absence of lipoxygenase, KTI, lectin, and 7S α′ subunit proteins)]. Among the 172 F_2_ seeds, 10 F_2_ seeds possessing tetra null alleles (*lox1lox2lox3*/*lox1lox2lox3*-*ti*/*ti*-*le*/*le*-*cgy1*/*cgy1*) were obtained and were planted in the greenhouse in July 2018. Young leaves from each of the 10 F_2_ plants grown in the greenhouse were used to screen the plant of the *rs2rs2* genotype based on DNA marker (Yang et al., 2014; Choi et al., 2017). Only two plants were selected, and two F_2_ plants were individually harvested. A random sample of 120 F_3_ seeds for each strain was planted on 8 July 2019 in a field at the university. One F_3_ strain was chosen based on plant type, maturation date, stem height, seed coat color, seed quality, seed weight, and yield. Then 360 random F_4_ seeds of the strain were planted in the university field on 9 July 2020. After harvesting, random F_5_ seeds were used to confirm recessive genotypes (*lox1lox2lox3*/*lox1lox2lox3*-*ti*/*ti*-*le*/*le*-*cgy1*/*cgy1*-*rs2*/*rs2*) by observing the absence of lipoxygenase, KTI, lectin, and 7S α′ subunit proteins. Low stachyose content (*rs2rs2* genotype) for the strain developed was confirmed by HPLC (Sung et al., 2014). The scheme for the improvement of penta null alleles (*lox1lox2lox3*/*lox1lox2lox3*-*ti*/*ti*-*le*/*le*-*cgy1*/*cgy1*-*rs2*/*rs2*) is presented in Figure 1.

### Determination of Lipoxygenase and 7S *α*′ Subunit Proteins by SDS-PAGE

Crude protein from the random F_5_ seeds of the breeding line and random seeds of the cultivar (“Daewon”) was obtained to identify the presence (“+”) or absence (“−” of lipoxygenase and 7S *α*′ subunit proteins. Lipoxygenase and 7S *α*′ subunit proteins were detected by sodium dodecyl sulfate-polyacrylamide gel electrophoresis (SDS-PAGE) performed according to Fling and Gregerson (1986). The cultivar (“Daewonkong”) was used as a control for the presence of lipoxygenase and 7S *α*′ subunit proteins (*Lox1Lox2Lox3*/*Lox1Lox2Lox3*-*Cgy1*/*Cgy1* genotype). Fine powder samples of the two materials were incubated for 30 min in 1 ml of Tris–HCl, pH 8.0, and 1.56% v/v β-mercaptoethanol. About 50 μl of the supernatant collected through centrifugation was added to an equivalent amount of 5× sample buffer containing 1 M Tris–HCl, pH 6.8, 50% v/v glycerol, 1.96% v/v β-mercaptoethanol, and 10% w/v sodium dodecyl sulfate (SDS). The sample obtained was boiled at 97°C for 5 min and centrifuged. About 2 μl of the supernatant was loaded on a 12% acrylamide SDS polyacrylamide gel electrophoresis medium from Owl Separation Systems Inc. (model: P9DS, Portsmouth, NH, United States). After electrophoresis at 120 V for 7 h, the gel was stained. The gel was then destained in destaining solution for several hours. A protein marker (Sigma Marker, Product Code: M4038) was used to identify the presence or absence of lipoxygenase protein (97 kDa) and 7S *α*′ subunit protein (72 kDa).

### Determination of KTI and Lectin Protein by Western Blot Analysis

Proteins obtained from the parental seed, each F_2_ seed, and random F_5_ seeds of the breeding line and cultivar (“Daewon”) were separated by 10% or 12% SDS-PAGE and transferred onto an Immobilon-P membrane (PVDF, Millipore). Western blot analysis for the KTI protein was performed as previously described (Krishnan et al., 2000; Krishnan, 2001). Preparation of the antibody and western blot for lectin protein analysis was performed according to a previous method (Vodkin and Raikhel, 1986). The cultivar (“Daewonkong”) was used as a control for the presence of KTI and lectin proteins (*TiTi*-*LeLe* genotype). After blocking for 2 h in TBS buffer containing 0.1% Tween 20, 20 mM Tris (pH 7.5), 150 mM NaCl, and 5% nonfat dried milk (Carnation, Glendale, CA), the membrane was incubated with the antibody of KTI and lectin protein for 1 h. The blot was incubated with a horseradish peroxidase conjugated secondary antibody after washing in TBS buffer. The complex was then visualized using an enhanced chemiluminescence kit (Amersham, Buckinghamshire, United Kingdom). The presence or absence of KTI and lectin proteins was determined visually. In F_2_ seed generation, the ratio of segregation for the presence or absence of KTI and lectin proteins was determined by Chi-square analysis.

### Determination of *rs2rs2* Genotype and Stachyose Component

Young leaves from each of the 10 F_2_ plants possessing tetra null alleles (*lox1lox2lox3*/*lox1lox2lox3*-*ti*/*ti*-*le*/*le*-*cgy1*/*cgy1*) growing in a greenhouse were used for DNA extraction to screen the plant of the *rs2rs2* genotype based on DNA marker. DNA from parental lines along with each F_2_ individual were isolated according to the protocol described by Saghai Maroof et al. (1984). To analyze markers, primers (Forward:5′-CGTGGAGCAGGTGTATGTGTGG-3′, Reverse:5′-GGCACCAGTCCAACT CCG TTAC-3′) were designed according to previous results (Dierking and Bilyeu, 2008). PCR for the genotype assay was carried out in a PTC-200 thermocycler (MJ Research/Bio-Rad, Hercules, CA, United States) with the following conditions: 95°C for 5 min followed by 29 cycles of 95°C for 20 s, 65°C for 20 s, 72°C for 30 s, and a final extension at 72°C for 5 min. PCR products were electrophoresed in 2.5% 0.5× TBE agarose gels and were stained with EtBr. Gels were photographed under transmitted UV light. Stachyose content for the breeding line and cultivar (“Daewonkong”) was analyzed using the high-performance liquid chromatography (HPLC) method (Sung et al., 2014). The cultivar (“Daewonkong”) was used as a control for the normal content of stachyose (*Rs2Rs2* genotype).

### Agronomic Traits of Penta Null Genotype

First, 360 random F_5_ seeds of the breeding line and seeds of a cultivar (“Daewonkong”) as a control were planted in the university field on 15 July 2021. The experimental field had a completely randomized design with three replications. The plots included four rows 3-m long spaced 0.65 m apart. The seeding rate was 30 seeds per row. The soil type was a silty clay loam. Soil K, Ca, Mg, and Na averaged 0.46, 8.84, 2.83, and 0.28 cmolc/kg, respectively. Soil pH was 6.8. Agronomic traits such as maturation date, stem height, number of pods per plant, number of seeds per plant, 100-seed weight, stachyose content, and yield were recorded for the F_5_ plant generation of the breeding line (penta null genotype). The mean values of stem height, number of pods per plant, number of seeds per plant, 100-seed weight, stachyose content, and yield were compared by Duncan’s multiple range test at the 5% level.

## Results

### Selection of F_2_ Seeds With KTI and Lectin Proteins Free

**Figure 2:**
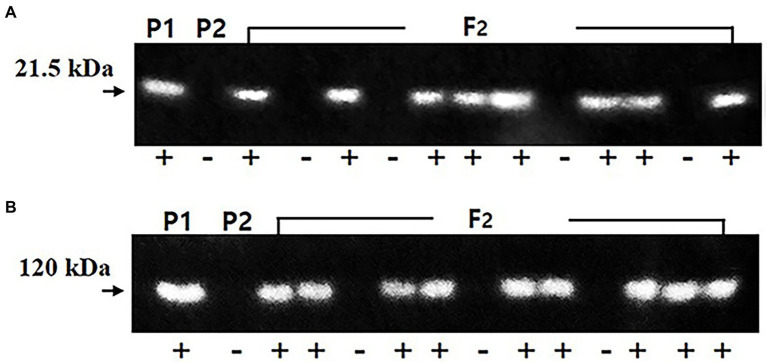
Segregation of KTI **(A)** and lectin **(B)** proteins in the parents and F_2_ seeds. Arrows indicate the KTI protein of 21.5 kDa and lectin protein of 120 kDa. P1: *lox1lox2lox3/lox1lox2lox3*-*Ti/Ti*-*Le/Le*-*cgy1/cgy1*-*rs2/rs2* genotype, P2: *lox1lox2lox3/lox1lox2lox3*-*ti/ti*-*le/le*-*cgy1/cgy1*-*Rs2/Rs2* genotype. +, −: presence and absence of KTI and lectin proteins.

A total of 172 F_2_ seeds were obtained from the cross of the *lox1lox2lox3*/*lox1lox2lox3*-*rs2*/*rs2*-*cgy1*/*cgy1* parent and *lox1lox2lox3* /*lox1lox2lox3*-*ti*/*ti*-*le*/*le*-*cgy1/cgy1* parent. Each seed was analyzed for the segregation of KTI and lectin proteins. KTI protein of 21.5 kDa and lectin protein of 120 kDa were segregated in the F_2_ seed generation (Figure 2). The segregation data for KTI and lectin proteins in the F_2_ seed generation are presented in Table 2.

**Table 2:** Segregation for the presence (+) or absence (−) of Kunitz trypsin inhibitor (KTI) and lectin proteins in F_2_ seed generation derived from the cross of a *lox1lox2lox3*/*lox1lox2lox3*-*rs2*/*rs2*-*cgy1*/*cgy1* parent and *lox1lox2lox3*/*lox1lox2lox3*-*ti*/*ti*-*le*/*le*-*cgy1/cgy1* parent.

KTI	Lectin	Seed number		*χ* ^2^ value (9:3:3:1)	*p*-value
		Observed	Expected		
Present	Present	102	96.75	2.883	0.5–0.1
Present	Absent	36	32.25		
Absent	Present	24	32.25		
Absent	Absent	10	10.75		

Among the 172 F_2_ seeds, 138 F_2_ seeds showed KTI protein and 34 F_2_ seeds did not show KTI protein. Lectin protein existed in 126 F_2_ seeds, and 46 F_2_ seeds did not show lectin protein. The segregation ratio for the presence or absence of KTI and lectin proteins in the F_2_ seed generation was fitted to an expected 3:1 ratio (*χ*^2^ = 2.51 for KTI and 0.28 for lectin proteins). Between KTI protein and lectin protein, the segregation ratios of 102 *Ti_Le_:* 36 *Ti_lele*: 24 *titiLe_*: 10 *titilele* were observed (*χ*^2^ = 2.883, *p* = 0.5–0.1). Ten F_2_ seeds possessing tetra null alleles (*lox1lox2lox3*/*lox1lox2lox3*-*ti*/*ti*-*le*/*le*-*cgy1*/*cgy1*) were selected and planted to select the plant in the greenhouse with the *rs2rs2* genotype based on the DNA marker.

### Selection of F_2_ Plants With *rs2rs2* Genotype Using DNA Marker

**Figure 3:**
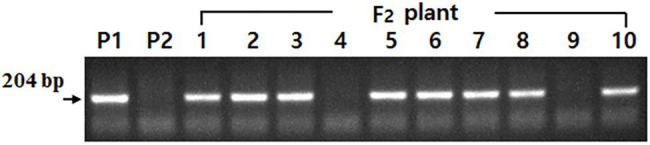
Segregation of DNA marker based on *Rs2* allele in parents and 10 F_2_ individual plants with the tetra null genotype (*lox1lox2lox3*/*lox1lox2lox3*-*ti*/*ti*-*le*/*le*-*cgy1*/*cgy1*). P1: *lox1lox2lox3/lox1lox2lox3*-*ti/ti*-*le/le*-*cgy1/cgy1*-*Rs2/Rs2* genotype. P2: *lox1lox2lox3/lox1lox2lox3*-*Ti/Ti*-*Le/Le*-*cgy1/cgy1*-*rs2/rs2* genotype. F_2_ plant—4 and 9; *rs2rs2* genotype.

The *rs2* allele-specific DNA marker showed segregation according to the individual plant in the F_2_ plant population consisting of 10 plants with the tetra null genotype (*lox1lox2lox3*/*lox1lox2lox3*-*ti*/*ti*-*le*/*le*-*cgy1*/*cgy1*). Among 10 F_2_ plants, eight showed a band (*Rs2_*genotype) and two showed no band (Figure 3). Thus, two F_2_ plants were identified as having the *rs2rs2* genotype. At maturity, two F_2_ plants identified as having the penta null genotype (*lox1lox2lox3*/*lox1lox2lox3*-*ti*/*ti*-*le*/*le*-*cgy1*/*cgy1*-*rs2/rs2*) were harvested separately. The F_3_ seeds obtained were used to advance F_5_ plant generation.

### Confirmation of Penta Null Line

**Figure 4:**
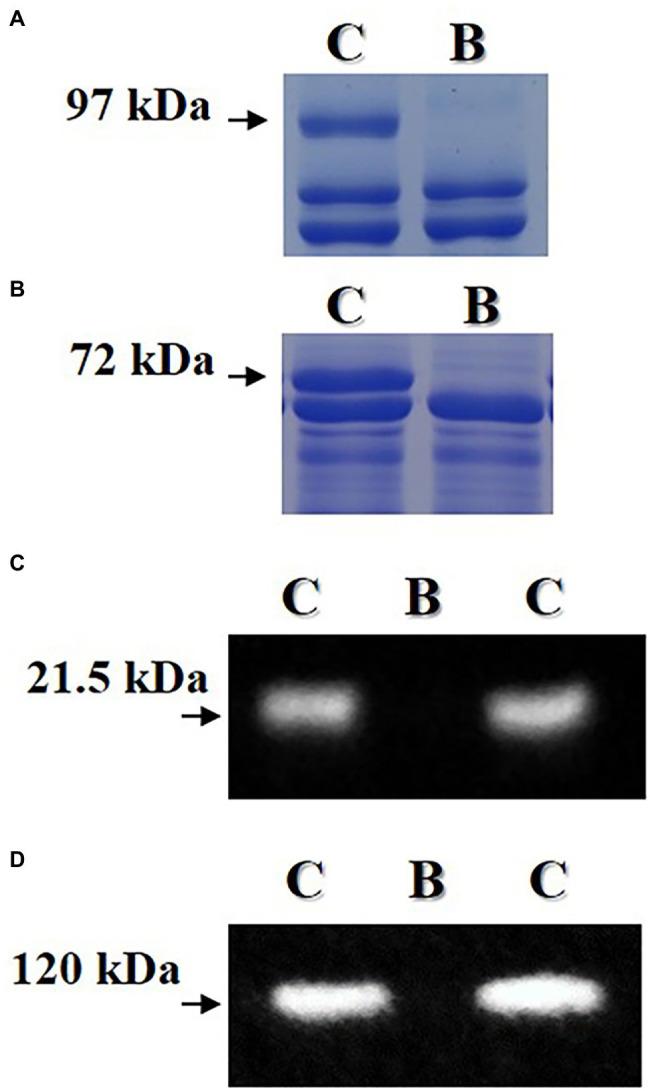
Confirmation of absence of lipoxygenase **(A)**, 7S α′ subunit **(B)**, KTI **(C)**, and lectin **(D)** proteins in mature seed of the breeding line (B) with the penta null genotype (*lox1lox2lox3*/*lox1lox2lox3*-*ti*/*ti*-*le*/*le*-*cgy*1/*cgy1*-*rs2*/*rs2*). Arrows indicate lipoxygenase protein of 97 kDa, 7S α′ subunit protein of 72 kDa, KTI protein of 21.5 kDa and lectin protein of 120 kDa. C: “Daewonkong” (*Lox1Lox2Lox3*/*Lox1Lox2Lox3*-*Ti*/*Ti*-*Le*/*Le*-*Cgy1*/*Cgy1-Rs2/Rs2* genotype.

Random F_5_ seeds for the breeding line were used to confirm the absence of lipoxygenase, KTI, lectin, and 7S *α*′ subunit proteins (Figure 4). Proteins of lipoxygenase and 7S *α*′ subunit were not observed in the mature F_5_ seed of the breeding line by using SDS-PAGE analysis. Also, proteins of KTI and lectin were not observed by using western blot analysis. However, these four proteins were observed in the seed of the “Daewonkong” (*Lox1Lox2Lox3* /*Lox1Lox2Lox3*-*Ti*/*Ti*-*Le*/*Le*-*Cgy1*/*Cgy1*-*Rs2/Rs2*) cultivar.

### Agronomic Traits of Penta Null Line

**Figure 5:**
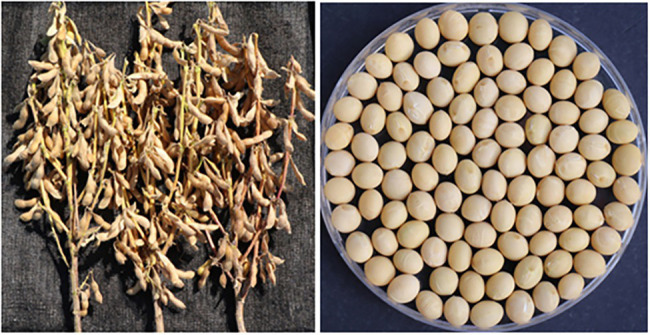
Appearance of F_5_ plants and F_6_ seeds possessing penta null alleles (*lox1lox2lox3*/*lox1lox2lox3*-*ti*/*ti*-*le*/*le*-*cgy1*/*cgy1-rs2/rs2*).

The breeding line has purple flowers, tawny pubescence, a determinate growth habit, and light yellow pods at maturity. Some quantitative traits of the breeding line are shown in Table 3. The breeding line matured on 16 October, which was 3 days later than “Daewonkong.” The stem height of the breeding line was 53.0 cm compared to the cultivar., “Daewonkong,” at 48.0 cm. The number of pods per plant and seeds per plant for the breeding line was 52 and 84, respectively. The stachyose content of the breeding line was 2.9 g/kg, which was much lower than 12.7 g/kg of the cultivar., “Daewonkong.” The 100-seed weight of the breeding line was 31.1 g, a little higher than that of “Daewonkong” (29.5 g). The yield of the breeding line was 2.80 t/ha, which was slightly higher than that of “Daewonkong” (2.78 t/ha). The plant type harvested and seeds of the breeding line with the penta null genotype (*lox1lox2lox3*/*lox1lox2lox3*-*ti*/*ti*-*le*/*le*-*cgy1*/*cgy1*-*rs2/rs2*) are shown in Figure 5. The seed of the breeding line has a yellow hilum and yellow seed coat color. The cotyledon color of the mature seed is yellow.

**Table 3:** Quantitative characteristics of the cultivar (“Daewonkong”) and breeding line strain in field conditions during 2021.

Cultivar/breeding line	Traits							
	Planting date	Maturing date	Stem height (cm)	NP/P	NS/P	Stachyose (g/kg)	SW (g)	Yield (Ton/ha)
“Daewonkong”	June 15	October 13	48.0^a^	55^a^	85^a^	12.7^a^	29.5^a^	2.78^a^
Breeding line	June 15	October 16	53.0^a^	52^a^	84^a^	2.9^b^	31.0^a^	2.80^a^

NP/P, number of pods per plant; NS/P, number of seeds per plant; SW, 100-seed weight. The same letters in the column are not significant at the 5% significance level by DMRT.

## Discussion

Demand for soybean and soybean products has increased in recent years because soybean is an excellent source of protein, oil, carbohydrates, and many other bioactive ingredients for humans. However, the lipoxygenase, KTI, lectin, 7S *α*′ subunit, and stachyose components that exist in the raw mature seeds of soybean are considered antinutritional and allergenic factors (Hata et al., 1991; Liener, 1995; Robinson et al., 1995; Katsuya et al., 2007; Krishnan et al., 2009). Heat treatment or other methods are needed to eliminate or reduce these components and secure the efficiency of nutrient absorption and food safety. But these treatments cause some changes and reduce soybean quality (Chen et al., 2019). Genetic removal or reduction of antinutritional and allergenic factors such as lipoxygenase, KTI, 7S α′ subunit, and stachyose components that exist in mature soybean seeds is needed to improve the nutritional values for the soybean food industry. Also, a cultivar with the penta null genotype (*lox1lox1lox2*/*lox2/ lox3*/*lox3*-*ti*/*ti*-*le*/*le*-*cgy*1/*cgy1*-*rs2*/*rs2*) for lipoxygenase, KTI, lectin, 7S α′ subunit, and stachyose components enhances the utilization of soybean in food as well as feed. A total of 172 F_2_ seeds were obtained from the cross of a *lox1lox2lox3*/*lox1lox2lox3*-*rs2*/*rs2*-*cgy1*/*cgy1* parent and *lox1lox2lox3* /*lox1lox2lox3*-*ti*/*ti*-*le*/*le*-*cgy1/cgy1* parent to produce seeds possessing penta null alleles (*lox1lox2lox3*/ *lox1lox2lox3*-*ti*/*ti*-*le*/*le*-*cgy1*/*cgy1*-*rs2*/*rs2*). KTI and lectin proteins were segregated in the F_2_ seed generation (Figure 2; Table 2). The segregation ratio for the presence or absence of KTI and lectin proteins was fitted to an expected 3:1 ratio (χ^2^ = 2.51 for KTI and 0.28 for lectin protein). This result substantiates previous observations that the presence or absence of KTI and lectin proteins is controlled by a single gene (Orf et al., 1978; Orf and Hymowitz, 1979). Segregation ratios of independent inheritance between KTI protein and lectin protein were observed (χ^2^ = 2.883, *p* = 0.5–0.1). This result was consistent with previous studies reporting that both *Ti* and *Le* alleles were independently inherited (Orf and Hymowitz, 1979; Moraes et al., 2006; Lee et al., 2008; Choi et al., 2021). *Ti* allele was independently inherited with the *Le* allele in the F_2_ population consisting of 24 plants (Moraes et al., 2006). Orf and Hymowitz (1979) observed that *Le* and *Ti* alleles were inherited independently by the F_2_ population with 96 plants. Lee et al. (2008) observed that *Ti* and *Le* alleles were independently inherited in 173 F_2_ seeds. Also, Choi et al. (2021) reported that *Le* and *Ti* alleles were independently inherited in F_2_ seed generation consisting of 210 seeds. Ten F_2_ seeds possessing tetra null alleles (*lox1lox2lox3*/ *lox1lox2lox3*-*ti*/*ti*-*le*/*le*-*cgy1*/*cgy1*) were obtained (Table 2). In a previous study, two F_2_ seeds possessing triple null alleles (*ti*/*ti*-*le*/*le*-*p34*/*p34*) were obtained from 150 F_2_ seeds (Schmidt et al., 2015). Also, three F_2_ seeds possessing tetra null alleles (*lox1lox2lox3*/*lox1lox2lox3*-*ti*/*ti*-*le*/*le*-*cgy1*/*cgy1*) were obtained from 210 F_2_ seeds (Choi et al., 2021). Among the 10 F₂ plants with the tetra null genotype (*lox1lox2lox3*/*lox1lox2lox3*-*ti*/*ti*-*le*/*le*-*cgy1*/*cgy1*), only two F₂ plants were selected by DNA marker (Figure 3). Its genotype was found to be *rs2rs2,* and it had low stachyose content compared to the normal cultivar with the *Rs2Rs2* genotype. The result that two individuals had the *rs2rs2* genotype in the F_2_ population of 10 individuals was consistent with the results of a previous study (Skoneczka et al., 2009; Yang et al., 2014). The absence of lipoxygenase, KTI, lectin, and 7S *α*′ subunit proteins was confirmed in random F_5_ seeds for the breeding line possessing the penta null genotype (*lox1lox2lox3*/*lox1lox2lox3*-*ti*/*ti*-*le*/*le*-*cgy1*/*cgy1*-*rs2/rs2*). However, in the “Daewonkong” (*Lox1Lox2Lox3* /*Lox1Lox2Lox3*-*Ti*/*Ti*-*Le*/*Le*-*Cgy1*/*Cgy1*-*Rs2/Rs2*) cultivar., these four proteins were observed (Figure 4). For the *rs2* allele, the *rs2rs2* genotype of the breeding line was confirmed by analysis of stachyose content using random F_5_ seeds (Table 3). The stachyose content of the breeding line was 2.9 g/kg, which was much lower than 12.7 g/kg of the check cultivar., “Daewon” (*Rs2Rs2* genotype). No negative effects on traits of field emergence, seed yield, maturity, height, and fatty acid content between lines derived from PI200508 containing the reduced stachyose content and wild types were reported (Neus et al., 2005). Song et al. (2018) reported that BSH-3 seeds with 7S α′ subunit protein-free accumulated high levels of free amino acids as compared with normal seeds, particularly arginine, and the amounts of several essential amino acids were significantly elevated in BSH-3 seeds. Rani et al. (2020) developed KTI-free soybean with normal protein content. Normal-protein KTI-free RILs were significantly higher in both acidic and basic subunit of glycinin and β-subunit of β-conglycinin fraction compared to low-protein KTI-free RILs. Kumar et al. (2021) developed 21 F_6_ KTI-free lines exhibiting 100-fresh seed weight > 45 g, sucrose content > 7%, and morphologically similar to vegetable soybean. Moisture content and pod yield of KTI-free lines at R6 stage were 64%–74.1% and 7.0–9.5 t∙ha^−1^, respectively. Genetic removal of lipoxygenase-2 improved the speed of emergence of sprouts and the length of the sprouts and sprouting, thereby enhancing the suitability of beans for sprouting. Genetic removal of the KTI gene did not have a significant effect on sprouting attributes, though it enhanced BBI concentration and improved protein digestibility (Kumar et al., 2022).

Quantitative traits of the breeding line with the penta null genotype are shown in Table 3. In spite of the absence of antinutritional and harmful immunological components such as lipoxygenase, KTI, lectin, 7S α′ subunit, and stachyose that exist in the mature soybean seed, the breeding line germinated, grew, flowered, and reproduced normally in the greenhouse and under field conditions when compared to the cultivar “Daewonkong” (Figure 5). Schmidt et al. (2015) reported that plants possessing triple null alleles (*ti*/*ti*-*le*/*le*-*p34*/*p34*) flowered and produced seeds without any overt differences in comparison with the standard “Williams 82″ cultivar. No significant differences were observed for stem height, number of pods per plant, number of seeds per plant, 100-seed weight, and yield between the breeding line with the penta null genotype and the “Daewonkong” cultivar. These results indicate that the penta null soybean line had no impact on these agronomic traits. These results suggest that stacking of recessive alleles for *Lox1*, *Lox2*, *Lox3*, *Ti*, *Le*, *Cgy1*, and *Rs2* genes results in a soybean cultivar with significantly reduced antinutritional and allergenic ingredients. This is the first soybean strain with the penta null (*lox1lox2lox3*/*lox1lox2lox3*-*ti*/*ti*-*le*/*le*-*cgy1*/*cgy1*-*rs2/rs2*) genotype (free of lipoxygenase, KTI, lectin, and 7S α′ subunit proteins, and low stachyose content). Since mature soybean seeds have more than 20 kinds of allergens, it is considered that some allergens are also present in the seeds of the breeding line with the penta null genotype obtained in this study. However, when compared to common soybean seeds, it seems that allergens are greatly reduced. The breeding line obtained through this study could be used as a valuable parent for the improvement of soybean cultivars that do not contain several antinutritional properties in mature seeds.

## Conclusion

Lipoxygenase, KTI, lectin, 7S α′ subunit, and stachyose components that exist in mature soybean seeds are considered antinutritional and allergenic factors. The objective of this study was to breed a new soybean strain with a penta null genotype (*lox1lox2lox3*/*lox1lox2lox3*-*ti*/*ti*-*le*/*le*-*cgy*1/*cgy1*-*rs2*/*rs2*) for these five components. A total of 172 F_2_ seeds were obtained from the cross of a *lox1lox2lox3*/*lox1lox2lox3*-*rs2*/*rs2*-*cgy1*/*cgy1* parent and *lox1lox2lox3*/*lox1lox2lox3*-*ti*/*ti*-*le*/*le*-*cgy1/cgy1* parent. Ten F_2_ seeds possessing tetra null alleles (*lox1lox2lox3*/*lox1lox2lox3*-*ti*/*ti*-*le*/*le*-*cgy1*/*cgy1*) were selected and two F_2_ plants with the *rs2rs2* genotype (low stachyose content) were developed. One F_3_ strain with proper agronomical traits was selected. Proteins of lipoxygenase, KTI, lectin, and 7S *α*′ subunit were not observed in the random mature F_5_ seeds of the breeding line with the penta null genotype. The penta null soybean line has purple flowers, a determinate growth habit, light yellow pod, yellow seed coat color, and yellow hilum. The breeding line matured on October 16. The stem height of the breeding line was 53.0 cm. The number of pods per plant and seeds per plant for the breeding line was 52 and 84, respectively. The stachyose content of the breeding line was 2.9 g/kg, which was much lower than 12.7 g/kg of the cultivar., “Daewonkong” (*Rs2Rs2* genotype). The 100-seed weight of the breeding line was 31.1 g. The yield of the breeding line was 2.80 t/ha.

## Data Availability Statement

The raw data supporting the conclusions of this article will be made available by the authors, without undue reservation.

## Author Contributions

SC, SL, JL, HO, SK, NK, and JC were involved in the experimental design, crossing, protein analysis, planting, harvesting, and data collection and interpretation as well as write-up of this research. All authors contributed to the article and approved the submitted version.

## Funding

This research was supported by the Basic Science Research Program through the National Research Foundation of Korea (NRF) funded by the Ministry of Education (NRF-2018R1D1A1B07045483) and Korea Institute of Planning and Evaluation for Technology in Food, Agriculture and Forestry (Research number: 119011-3).

## Conflict of Interest

The authors declare that the research was conducted in the absence of any commercial or financial relationships that could be construed as a potential conflict of interest.

## Publisher’s Note

All claims expressed in this article are solely those of the authors and do not necessarily represent those of their affiliated organizations, or those of the publisher, the editors and the reviewers. Any product that may be evaluated in this article, or claim that may be made by its manufacturer, is not guaranteed or endorsed by the publisher.
